# Associations Between Patterns of Alcohol Use and Viral Load Suppression Amongst Women Living with HIV in South Africa

**DOI:** 10.1007/s10461-021-03263-3

**Published:** 2021-04-19

**Authors:** B. Myers, C. Lombard, J. A. Joska, F. Abdullah, T. Naledi, C. Lund, P. Petersen Williams, D. J. Stein, K. R. Sorsdahl

**Affiliations:** 1 Alcohol, Tobacco and Other Drug Research Unit, South African Medical Research Council, Tygerberg, PO Box 19070, Cape Town, 7505 South Africa; 2 Department of Psychiatry & Mental Health, University of Cape Town, Cape Town, South Africa; 3 Biostatistics Unit, South African Medical Research Council, Cape Town, South Africa; 4 Office of AIDS and TB Research, South African Medical Research Council, Pretoria, South Africa; 5 Dean’s Office, Faculty of Health Sciences, University of Cape Town, Cape Town, South Africa; 6 School of Public Health & Family Medicine, University of Cape Town, Cape Town, South Africa; 7 Department of Psychiatry & Mental Health, Alan J Flisher Centre for Public Mental Health, University of Cape Town, Cape Town, South Africa; 8 South African Medical Research Council’s Unit on Risk & Resilience in Mental Disorders, Cape Town, South Africa

**Keywords:** Women, Alcohol, Viral load suppression, Mediators, HIV, South Africa

## Abstract

This study aimed to identify alcohol use patterns associated with viral non-suppression among women living with HIV (WLWH) and the extent to which adherence mediated these relationships. Baseline data on covariates, alcohol consumption, ART adherence, and viral load were collected from 608 WLWH on ART living in the Western Cape, South Africa. We defined three consumption patterns: no/light drinking (drinking ≤ 1/week and ≤ 4 drinks/occasion), occasional heavy episodic drinking (HED) (drinking > 1 and ≤ 2/week and ≥ 5 drinks/occasion) and frequent HED (drinking ≥ 3 times/week and ≥ 5 drinks/occasion). In multivariable analyses, occasional HED (OR 3.07, 95% CI 1.78–5.30) and frequent HED (OR 7.11, 95% CI 4.24–11.92) were associated with suboptimal adherence. Frequent HED was associated with viral non-suppression (OR 2.08, 95% CI 1.30–3.28). Suboptimal adherence partially mediated the relationship between frequent HED and viral non-suppression. Findings suggest a direct relationship between frequency of HED and viral suppression. Given the mediating effects of adherence on this relationship, alcohol interventions should be tailored to frequency of HED while also addressing adherence.

## Introduction

Despite the scale up of evidence-based HIV treatment, South African women continue to be disproportionately affected by HIV—comprising about 60% of the estimated 7.9 million South Africans living with HIV [1]. Although access to antiretroviral therapy (ART) has dramatically reduced HIV morbidity and mortality [2, 3], only about 69% of South African adult women living with HIV (WLWH) are virally suppressed [1]. As viral non-suppression increases the likelihood of rapid disease progression and onward transmission to sexual partners [4, 5], strategies are needed to improve rates of viral suppression among WLWH in South Africa [1].

South Africa, a low-and-middle income country (LMIC) at the southern tip of Africa, is known for its high volume of per capita alcohol consumption amongst people who drink. Although a substantial proportion (> 50%) of the adult population abstain from using alcohol, heavy episodic drinking (HED) is the normative pattern of alcohol use amongst people who drink [6, 7]. HED is sometimes referred to as binge drinking and is typically defined as the consumption of five or more standard drinks or 60 g of absolute alcohol during a single occasion [7]. Similar patterns of alcohol use have been reported amongst people on ART- South African studies estimate that 40% to 63% of PLWH on ART who drink do so at hazardous or harmful levels [8–11]. Comparable rates of HED have been reported for women on ART, ranging between 30 and 75% of current drinkers [8–10]. These patterns of alcohol consumption are of grave concern given evidence from systematic reviews that high levels of alcohol consumption are associated with poorer engagement and retention in HIV care and lower rates of ART adherence for PLWH in both high income countries [12, 13] and in LMICs within sub-Saharan Africa [14]. While some studies also report associations between alcohol use and viral non-suppression, the nature of the relationship between alcohol use and viral suppression requires further clarification [15, 16].

Prior studies examining the relationship between alcohol consumption and viral suppression have produced inconsistent results. Some studies found no association between alcohol use and viral suppression after controlling for self-reported ART adherence, suggesting an indirect relationship with viral suppression explained by alcohol’s association with adherence [17–19]. Other studies have reported associations between alcohol use and viral suppression after controlling for adherence [20–23]. This raises the possibility of alternative, possibly biological pathways through which alcohol may impact response to HIV treatment. Further, only two studies used mediation analysis to examine these relationships which limits our understanding of the importance of the adherence pathway relative to other potential pathways. These studies produced conflicting results: one reported direct effects of alcohol use on viral suppression and indirect effects via adherence [23], whereas the other only found indirect effects through adherence [20]. Both these studies were conducted in the US. Additional research is needed to examine whether findings from these studies can translate to South Africa where the prevalence of HIV is greater, the degree of epidemiological control is poorer, and where risk is generalized rather than concentrated among racial and sexual minorities [1, 4]. In addition, participants in these two studies were predominantly male. As research has demonstrated sex differences in physiological responses to alcohol [24], the extent to which alcohol’s association with viral suppression is mediated by adherence for WLWH requires clarification [16, 17, 25].

Measurement imprecision may explain some of these inconsistent findings. Studies have used a wide range of alcohol consumption measures (quantity only, alcohol use screening tools, frequency only), making it difficult to compare findings across studies [23, 26]. In addition, most prior studies did not examine how specific patterns of drinking relate differently to viral suppression. An exception has been Cook et al. [23] who specified patterns of drinking characterised by different degrees of frequency and quantity, noting that frequent heavy drinking impacted on viral suppression, but not other patterns of drinking marked by occasional heavy drinking, light drinking, or abstinence. However, there is relatively little information on how these alcohol consumption patterns are associated with viral suppression among WLWH, and the extent to which this relationship is mediated by ART adherence [25].

Addressing this information gap is important for the design of interventions aimed at improving rates of viral suppression among WLWH, such as the South African Medical Research Council’s Social Impact Bond for Adolescent Girls and Young Women [27]. If alcohol consumption patterns characterized by abstinence or light drinking are associated with greater likelihood of ART adherence and viral suppression, then interventions that promote abstinence or very limited use of alcohol may be indicated. Furthermore, evidence that certain drinking patterns infer greater risk for poor adherence and viral suppression could identify WLWH who may benefit most from these interventions. If findings show that adherence fully mediates the relationship between drinking and viral suppression, interventions focused on identifying and reducing barriers to adherence during periods of alcohol use would be needed. In contrast, if findings show that adherence only partially mediates alcohol’s association with viral suppression, interventions that focus on reducing alcohol use and facilitating ART adherence would be required.

This paper aims to provide this information by examining (i) the associations between a range of alcohol consumption patterns and HIV viral suppression and (ii) the extent to which these associations are mediated by differences in adherence to ART among a cohort of women obtaining treatment for HIV from primary care clinics in the Western Cape province of South Africa.

## Methods

This paper analyses baseline data on women receiving treatment for HIV who participated in Project MIND—a three-arm cluster randomized controlled trial comparing the effectiveness of different resourcing approaches to integrating counselling for unhealthy alcohol use and depression into HIV care (Trial Registration Number: PACTR201610001825403). Trial methods are described in detail elsewhere [27].

### Study Setting, Participants, and Procedures

From May 2017 to March 2019, 608 WLWH were recruited from 24 primary care clinics. offering free HIV treatment services to patients from geographically distinct catchment areas. Located in four of the province’s six health districts, 15 of these clinics served urban communities and nine served rural communities [28].

During the recruitment period, HIV care providers asked all patients presenting for routine HIV treatment at these clinics about their recent alcohol use and mood. Individuals reporting alcohol use or low mood were referred to a study assessor for eligibility screening. Patients who were at least 18 years old and taking ART for HIV; obtained an Alcohol Use Disorders Identification Test score ≥ 8 (indicating hazardous alcohol use) [10] or a Center for Epidemiologic Studies Depression Scale score ≥ 16 (indicating possible depression) [29] were eligible to participate provided they were not receiving other mental health treatment or participating in another study. Eligible patients who were interested in study participation were enrolled into the study. Recruitment was balanced across these clinics, with participant enrolment continuing until 25 participants (irrespective of gender) meeting eligibility criteria for alcohol and 25 participants meeting eligibility criteria for depression had been enrolled from each clinic.

At the enrolment appointment, an assessor obtained the patient’s written informed consent for trial participation prior to conducting a computer-assisted personal interview in English, Afrikaans. or isiXhosa (the three official languages of the province). Interviews included questions on socio-demographic characteristics, HIV treatment, alcohol use, common mental disorders, and health service use. Participants also provided whole blood samples for HIV viral load testing which were sent to an accredited pathology laboratory for analysis. All study activities occurred in private rooms at the participating clinic.

### Measures

#### HIV Viral Suppression

HIV viral load values were coded into three categories: virally suppressed (< 40 copies/ml); unsuppressed (40–999 copies/ml), and unsuppressed with possible treatment failure (≥ 1000 copies/ml). To ensure adequate power, we re-coded these values into two categories: viral suppression (< 40 copies/ml) and non-suppression (≥ 40 copies/ml).

#### Patterns of Alcohol Use

To examine frequency of alcohol consumption, participants were asked to recall the number of days they drank alcohol in the month preceding the baseline assessment. Assessors used a calendar to assist participants in recalling days they used alcohol. Using this information, we calculated the average number of drinking days on a typical week. Overall participants drank on average 1.0 days per week in the 30 days preceding the assessment (95% CI 0.8–1.1), but responses were skewed with 43.4% (95% CI 38.7–48.2) of the sample reporting no alcohol consumption over this time. Guided by the distribution of responses and the literature on meaningful categories of weekly drinking frequency [29, 30], we recoded this variable into four categories of weekly drinking frequency: abstinence (n = 264; 43.4% of participants); drinking ≤ 1 day per week (n = 151; 24.8%); drinking > 1 day and ≤ twice per week (n = 100; 16.5%); and drinking ≥ three times per week (n = 93; 15.2%).

Quantity of standard alcoholic drinks consumed on a typical drinking day was assessed by a single questionnaire item, with categorical response options ranging from 0, 1–2 drinks, 3–4 drinks, 5–6 drinks, 7–9 drinks or 10 or more drinks. As we were interested in examining typical patterns of alcohol consumption in the South African context (notably abstinence and HED), we recoded these response options into abstinence (0 drinks), no HED (≥ 1 and < 5 standard drinks typically consumed) and HED (≥ 5 drinks). Although most studies define HED for women as either 40 g or 60 g of absolute alcohol on a single occasion [7, 30], we chose the higher threshold (60 g of absolute alcohol amounts to ≥ five standard drinks) as this is more commonly used in South Africa [6]. To improve participants’ estimations of the number of standard drinks consumed, a challenge in South Africa where people drink a combination of formally and informally produced alcohol [7, 31, 32], participants were shown pictures of frequently consumed alcoholic beverages in this setting.

In an effort to replicate Cook et al.’s [23] approach and allow for meaningful comparisons, and guided by recent literature demonstrating that patterns of alcohol use representing varying frequency and quantity of alcohol consumption have differential effects on health outcomes [30], we used these two indicators to define four past-month alcohol consumption patterns: no drinking (abstainers), light drinking (≤ once per week and ≤ four drinks per occasion reflecting no HED), occasional HED (drinking once or twice a week and ≥ five drinks per occasion) and frequent HED (drinking three or more times per week and ≥ five drinks per occasion). As only 58 women reported light drinking, we combined this with the “no drinking” category. Sensitivity analyses (not reported here) found that combining these categories had little effect on the results.

#### ART Adherence

ART adherence was assessed by self-report. Self-reported adherence is strongly associated with HIV outcomes and is simpler to obtain than estimates from pill counts or biomarkers [33]. We used the Center for Adherence Support Evaluation (CASE) adherence index [33, 34], a scale that has been widely used in sub-Saharan Africa to measure adherence to ART [34–36]. This scale is a composite of three self-reported indicators of adherence, namely: frequency of “difficulty in taking HIV medications on time”, “average number of days per week that at least one dose of HIV medications were missed”, and “last time missed one dose of HIV medications” [34]. Responses on these items are summed to produce a total adherence score ranging from 3 to 16. Higher scores indicate better adherence, with score > 10 indicative of good adherence. Scores > 10 have been shown to correlate with self-reported adherence rates of 95% and viral suppression [34].

#### Covariates

Covariates included the socio-demographic variables of age (18–30, 31–39, 40–49, ≥ 50 years of age), education (completed high school vs. did not complete high school), relationship status (partner vs. no partner), unemployment, and average monthly income (≤ R2000; R2001–R4000; ≥ R4000; at the time of the study USD 1 ~ ZAR 15). HIV-related covariates included number of years since HIV diagnosis and number of years on ART. Psychosocial covariates included probable depression as assessed by the CES-D where a score of 16 or higher is suggestive of probable depression [28]. Perceived social support was assessed using the 19-item Medical Outcomes Study (MOS) Social Support Survey, with higher composite scores indicative of more social support [37]. A single item explored any illicit drug use in the past year, with responses coded as yes or no.

### Analyses

All analyses were conducted using Stata 16 (Stata Corporation, College Station, TX, USA), with significance set at *p* < 0.05. We used Stata’s survey analysis platform to account for clustering. Using available case analysis to address missing data, a total of 608 observations were included. First, we conducted adjusted Wald test (for categorial variables) and linear regression (for continuous variables) analyses to identify and describe possible relationships between risk factors and the three categories of viral response to ART (suppressed, non-suppressed, non-suppressed with possible treatment failure). We examined the distribution of cases across the three viral load categories for each pattern of alcohol use (none/light, occasional HED, frequent HED). Due to small cell sizes, we dichotomised viral load results into suppressed or non-suppressed categories for all further analyses. Second, we ran logistic regression and adjusted Wald test analyses (for categorial variables) and performed linear regression (for continuous variables) to identify possible relationships between covariates and the hypothesized mediator (adherence) and the exposure of interest (patterns of drinking). Variables associated with drinking patterns and adherence at *p* < 0.10 were entered in multinomial logistic and multivariable logistic regression models.

Third, we used logistic regression to explore whether the exposure of interest and hypothesised mediator were associated with the outcome of interest. Variables associated with viral suppression at *p* < 0.10 were entered in a multivariable model. This enabled us to examine the independent effects of adherence and drinking patterns on viral non-suppression while adjusting for potential confounders. We examined possible interactions between age and alcohol consumption patterns use as well as alcohol consumption patterns and adherence in separate regression models.

Finally, we used generalised structural equation modelling (GSEM) to examine the potentially mediating effects of (i) adherence on associations between drinking patterns and viral non-suppression and (ii) drinking patterns on the relationship between age and viral non-suppression while adjusting for other covariates. GSEM allows for the analysis of categorical data using non-linear assumptions for modelling dichotomous data and provides a statistical test of coefficients from mediation analyses [38, 39]. Results are presented graphically, with beta coefficients (*b*) and standard errors (SE) reported for each path. As GSEM is not able to decompose the total effects into indirect and direct effects for categorical data, we used *ldecomp* to calculate estimates and confidence intervals of the direct and indirect effects of drinking patterns on viral suppression using bootstrap methods that accounted for clustering [40]. The indirect effect quantifies the portion of the total effect that is mediated by adherence and the direct effect represents the portion of the total effect that is not explained by the mediator.

## Results

### Demographic and Clinical Characteristics of the Sample

The demographic and clinical characteristics of the sample are presented in Table 1. Most women in the sample were single (71.7%), had not completed high school (87.3%), were unemployed (65.6%), with a monthly income below R2000 (78.0%). Women ranged between 20 and 62 years of age (M = 38.5, SD = 10.0). A quarter of the sample were between 18 and 30 years old. On average, participants had been using ART for five years (M = 5.6, SD = 4.5); 35.7% reported suboptimal adherence. Most participants scored above the cut-off for probable depression (85.0%). Just over half the sample (53.0%) reported no or light drinking in the past month, 32.9% reported occasional HED and 14.1% reported frequent HED. Only 2.6% of the sample reported past year illicit drug use. Viral suppression was achieved by 64.6% of the sample (Table 1). Notably, 110 participants (18.1%) were at risk of treatment failure, of whom 60 had viral loads ≥ 10 000 copies/ml, indicative of high infectiousness. In bivariate analyses, only age (F(6, 10) = 4.87; p = 0.004), ART adherence (F(2, 22) = 7.53; p = 0.003), and patterns of alcohol use (F(4, 20) = 6.86; p = 0.001) were significantly associated with viral load categories (Table 1).

**Table 1:** Baseline characteristics for the total sample and by viral load status for women living with HIV in the MIND cohort (n = 608)

Variable	Total sample (N = 608) n (%)	HIV viral load categories				
		Viral load < 40 copies/ml (N = 393) n (%)	Viral load 40–999 copies/ml (N = 105) n (%)	Viral load ≥ 1000 copies/ml (N = 110) n (%)	Test statistic^a^	p value
Covariates						
Age categories						
18–30	152 (25.0%)	87 (57.2%)	31 (20.4%)	34 (22.4%)	F(6, 10) = 4.87	0.004
31–39	185 (30.4%)	111 (60.0%)	32 (27.3%)	42 (22.7%)		
40–49	175 (28.8%)	121 (69.1%)	26 (14.9%)	28 (16.0%)		
≥ 50	96 (15.8%)	76 (79.2%)	14 (14.6%)	6 (6.3%)		
Relationship						
Partner	172 (28.3%)	112 (65.1%)	27 (15.7%)	33 (19.2%)	F(2, 22) = 0.24	0.788
No partner	436 (71.7%)	283 (64.9%)	76 (17.4%)	77 (17.7%)		
Completed high school	77 (12.7%)	56 (72.7%)	11 (12.9%)	10 (13.0%)	F(2, 22) = 1.38	0.270
Unemployed	399 (65.6%)	247 (61.9%)	71 (17.8%)	81 (20.3%)	F(2, 22) = 2.80	0.082
Monthly income						
≤ R2000	474 (78.0%)	300 (63.3%)	83 (17.5%)	91 (19.2%)	F(4, 20) = 1.64	0.204
R2001–R4000	113 (18.6%)	81 (71.7%)	17 (15.0%)	15 (13.3%)		
≥ R4000	21 (3.5%)	14 (66.7%)	3 (14.3%)	4 (19.0%)		
Years living with HIV (M, SD)	7.4 (5.2)	7.31 (5.2)	7.35 (5.04)	7.91 (5.04)	F(2, 22) = 0.79	0.467
Years on ART (M, SD)	5.6 (4.5)	5.51 (4.5)	5.41 (4.25)	6.05 (4.71)	F(2, 22) = 0.89	0.423
Past year illicit drug use	16 (2.6%)	8 (50.0%)	4 (25.0%)	4 (25.0%)	F(2, 22) = 0.62	0.546
Probable depression	517 (85.0%)	341 (65.9%)	86 (16.6%)	90 (17.4%)	F(2, 22) = 0.68	0.517
Social support scale (M, SD)	68.2 (21.1)	67.8 (21.0)	70.1 (20.4)	67.5 (22.4)	F(2, 22) = 0.50	0.611
Hypothesised mediator						
CASE optimal ART adherence	391 (64.3%)	275 (70.3%)	56 (14.3%)	60 (15.4%)	F(2, 22) = 7.53	0.003
CASE suboptimal ART adherence	217 (35.7%)	120 (55.3%)	47 (21.7%)	50 (23.0%)		
Exposure of interest: Past month patterns of alcohol use						
No or light drinking	322 (53.0%)	221 (68.6%)	45 (14.0%)	56 (17.4%)	F(4, 20) = 6.86	0.001
Occasional HED	200 (32.9%)	137 (68.5%)	34 (17.0%)	29 (14.5%)		
Frequent HED	86 (14.1%)	37 (43.0%)	24 (27.9%)	25 (29.1%)		

ART antiretroviral therapy, *HED* heavy episodic drinking, defined as drinking five or more standard drinks on one occasion

^a^Adjusted Wald tests were conducted for categorical variables and linear regression analyses were conducted for continuous variables

### Covariates Associated with the Exposure of Interest and Hypothesised Mediator

In the multinomial logistic regression model examining variables associated with past month drinking patterns (Table 2), women 31 to 39 years of age had quadruple the odds of reporting frequent HED (RRR 4.40; 95% CI 1.75–10.89) than women ≥ 50 years of age. There was a trend towards women 18 to 30 years old being more likely to report frequent HED than women ≥ 50 years of age, but this was non-significant (RRR 3.21; 95% CI 0.95–10.85). Women who were unemployed were more likely to report frequent HED (OR 2.66; 95% CI 1.59–4.44) than those who were employed. Women with probable depression were less likely to report occasional HED (OR 0.11; 95% CI 0.05–0.23) or frequent HED (OR 0.13; 95% CI 0.05–0.30) than women without this comorbidity.

**Table 2:** Univariate and multivariate analyses of factors associated with past month patterns of alcohol use among women living with HIV in the MIND cohort (n = 608)

Variables	Univariate associations^a^				Multivariate model^b^	
	No or light drinking N = 322 n (%)	Occasional HED N = 200 n (%)	Frequent HED N = 86 n (%)	Test statistic; p value	Occasional HED RRR (95% CI)	Frequent HED N = 86 RRR (95% CI)
Covariates						
Age groups (years)						
18–30	69 (45.4%)	60 (39.5%)	23 (15.1%)	F (6, 18) = 2.66; p = 0.050	1.80 (0.93–3.50)	3.21 (0.95–10.85)
31–39	92 (49.7%)	55 (29.7%)	38 (20.5%)		1.42 (0.66–3.04)	4.40 (1.75–10.89)
40–49	98 (56.0%)	58 (33.1%)	19 (10.9%)		1.30 (0.67–2.48)	1.94 (0.84–4.51)
≥ 50	63 (65.6%)	27 (28.1%)	7 (6.3%)		Reference	
Relationship						
Partner	101 (58.7%)	52 (30.2%)	19 (11.1%)	F (2, 22) = 2.02; p = 0.156	-	-
No partner	221 (50.7%)	148 (33.9%)	67 (50.4%)		-	-
Completed high school	46 (59.7%)	22 (28.6%)	9 (11.7%)	F (2, 22) = 0.70; p = 0.509	-	-
Unemployed	204 (51.1%)	125 (31.3%)	70 (17.5%)	F (2, 22) = 8.32; p = 0.002	1.08 (0.63–1.86)	2.66 (1.59–4.44)
Monthly income						
≤ R2000	252 (53.2%)	148 (31.2%)	74 (15.6%)	F (4, 20) = 1.54; p = 0.227	-	-
R2001–R4000	60 (53.1%)	42 (37.2%)	11 (9.7%)		-	-
≥ R4000	10 (47.6%)	10 (47.6%)	1 (4.8%)		-	-
Past year illicit drug use	7 (43.8%)	4 (25.0%)	5 (31.3%)	F (2, 22) = 1.21; p = 0.318	-	-
Probable depression	309 (59.8%)	143 (27.7%)	65 (12.6%)	F (2, 22) = 15.05; p < 0.001	0.11 (0.05–0.23)	0.13 (0.05–0.30)
Social support (M, SD)	66.9 (21.6)	70.0 (20.6)	68.7 (20.6)	F (2, 22) = 0.81; p = 0.442	-	-

*HED* heavy episodic drinking, defined as drinking five or more drinks on one occasion, *RRR (95% CI)*  relative risk ratios and 95% confidence intervals

^a^Adjusted Wald tests were conducted for categorical variables and linear regression analyses were conducted for continuous variables

^b^Multinomial regression analysis was conducted with no or light drinking as the base outcome

In the multivariable logistic regression model of factors associated with suboptimal adherence (Table 3), women who reported past-year illicit drug use (aOR 3.63; 95% CI 1.76–7.48), probable depression (aOR 1.85; 95% CI 1.14–3.01), occasional HED (aOR 3.07; 95% CI 1.78–5.30) or frequent HED (aOR 7.11; 95% CI 4.24–11.92) had significantly greater odds of suboptimal ART adherence than those without these exposures.

**Table 3:** Univariate and multivariate analyses of factors associated with suboptimal ART adherence among women living with HIV in the MIND cohort (n = 608)

Variables	Univariate associations^a^			Multivariate model^b^
	Sub-optimal adherence (N = 217) n (%)	Optimal adherence (N = 391) n (%)	Test statistic; p value	aOR (95% CI)
Covariates				
Age categories (years)				
18–30	65 (42.8%)	87 (57.2%)	F (3, 21) = 1.66; p = 0.206	–
31–39	68 (36.8%)	117 (63.2%)		–
40–49	58 (33.1%)	117 (66.9%)		–
≥ 50	26 (27.1%)	70 (72.9%)		–
Relationship				
Partner	60 (34.9%)	112 (65.1%)	F (1, 23) = 0.04; p = 0.836	–
No partner	157(36.0%)	279 (67.0%)		–
Completed high school	24 (31.2%)	53 (68.8%)	F (1, 23) = 0.08; p = 0.383	–
Unemployed	141 (35.3%)	133 (64.7%)	F (1, 23) = 0.03; p = 0.886	–
Monthly income				
≤ R2000	173 (36.5%)	301 (63.5%)	F (2, 22) = 3.04; p = 0.068	Reference
R2001–R4000	34 (30.1%)	79 (69.9%)		0.78 (0.55–1.10)
≥ R4000	10 (47.6%)	11 (52.4%)		1.75 (0.56–5.45)
Past year illicit drug use	10 (62.5%)	6 (37.5%)	F (1, 23) = 4.96; p = 0.036	3.63 (1.76–7.48)
Probable depression	332 (64.2%)	185 (35.8%)	F (1, 23) = 0.02; p = 0.886	1.85 (1.14–3.01)
Social support scale (M, SD)	66.8 (19.8)	68.9 (21.8)	F (1, 23) = 0.85; p = 0.369	–
Exposure: Past month patterns of alcohol use No/light	74 (23.0%)	248 (77.0%)	F (2, 22) = 30.67; p < 0.001	Reference
Occasional HED	87 (43.5%)	113 (56.5%)		3.07 (1.78–5.30)
Frequent HED	56 (65.1%)	30 (34.9%)		7.11 (4.24–11.92)

^a^Adjusted Wald tests were conducted for categorical variables and linear regression analyses were conducted for continuous variables

^b^Multivariable logistic regression analysis was conducted with optimal adherence as the reference outcome

*aOR (95% CI)* adjusted odds ratios and 95% confidence intervals, *HED* heavy episodic drinking, defined as drinking five or more drinks on one occasion

### Variables Associated with Viral Non-suppression

In multivariate logistic regression analyses of viral non-suppression (Table 4), women between 18 to 30 and 31 to 39 years of age had more than double the odds of being non-suppressed relative to women ≥ 50 years of age (OR 2.51; 95% CI 1.32–4.78 and OR 2.23; 95% CI 1.13–4.41, respectively). Suboptimal ART adherence was associated with significantly greater odds of being virally non-suppressed (OR 1.75; 95% CI 1.20–2.57). Frequent HED but not occasional HED was associated with greater odds of being virally non-suppressed (OR 2.08; 95% CI 1.30–3.28) relative to no/light drinking. We found no evidence of significant interactions between age and drinking patterns or drinking patterns and adherence.

**Table 4:** Univariate and multivariate associations between past month patterns of alcohol use, antiretroviral adherence and HIV viral non-suppression among women living with HIV in the MIND cohort (n = 608)

Characteristic	Univariate logistic regression		Multivariate logistic regression	
	OR^1^	95% CI^2^	aOR^3^	95% CI
Covariates				
Age categories				
Reference: ≥ 50				
18–30	3.46	1.57–5.12	2.51	1.32–4.78
31–39	2.83	1.33–4.83	2.23	1.13–4.41
40–49	1.96	1.00–2.87	1.70	0.94–3.10
Relationship				
Partner	0.99	0.65–1.51	–	–
Completed high school	0.66	0.34–1.28	–	–
Unemployed	1.49	0.97–2.10	1.29	0.85–1.94
Monthly income				
Reference: ≥ R4000				
≤ R2000	1.16	0.47–2.88	–	–
R2001-R4000	0.79	0.27–2.31	–	–
Years living with HIV	1.01	0.99–1.04	–	–
Years on ART^4^	1.01	0.97–1.06	–	–
Past year illicit drug use	1.88	0.66–5.40	–	–
Probable depression	0.75	0.48–1.17	0.78	0.46–1.32
Social support scale	1.00	0.99–1.01	–	–
Hypothesised mediator				
CASE suboptimal adherence	1.92	1.38–2.65	1.75	1.20–2.57
Exposure: Patterns of alcohol use				
Reference: No/light drinking				
Occasional HED^5^	1.01	0.65–1.57	0.84	0.53–1.33
Frequent HED	2.90	1.98–4.25	2.08	1.30–3.28
Interaction: Age*Drinking pattern				
18–30* Occasional HED				0.55 (0.11–2.79)
18–30* Frequent HED				0.37 (0.06–2.34)
31–39* Occasional HED				0.60 (0.10–3.73)
31–39* Frequent HED				1.69 (0.25–11.36)
40–49* Occasional HED				1.32 (0.28–6.26)
40–49* Frequent HED				2.36 (0.29–19.14)
Interaction: Adherence* Drinking pattern				
Sub-optimal adherence*Occasional HED				0.78 (0.30–2.07)
Sub-optimal adherence*Frequent HED				1.71 (0.64–4.58)

*OR* odds ratio, *CI* confidence interval, *aOR* adjusted odds ratio; all variables associated with suppression at p < 0.1 entered into the model, *ART *  antiretroviral therapy, *HED*heavy episodic drinking, defined as drinking five or more drinks on one occasion

### Results of Mediation Analyses

Findings show significant associations between occasional HED (*β* = 0.24, *SE* = 0.06, *p* < 0.001) and frequent HED (*β* = 0.45, *SE* = 0.05, *p* < 0.001) and suboptimal ART adherence. Depression was also associated with suboptimal adherence *(β* = 0.13, *SE* = 0.05, *p* = 0.014). In turn, suboptimal adherence was associated with HIV viral non-suppression (*β* = 0.57, *SE* = 0.18; *p* = 0.010). Frequent HED (*β* = 0.71, *SE* = 0.22, *p* = 0.005) but not occasional HED (*β* = -0.23, *SE* = 0.24, *p* = 0.335) or depression (*β* = -0.25, *SE* = 0.25, *p* = 0.329) was significantly associated with viral non-suppression.

**Fig. 1:**
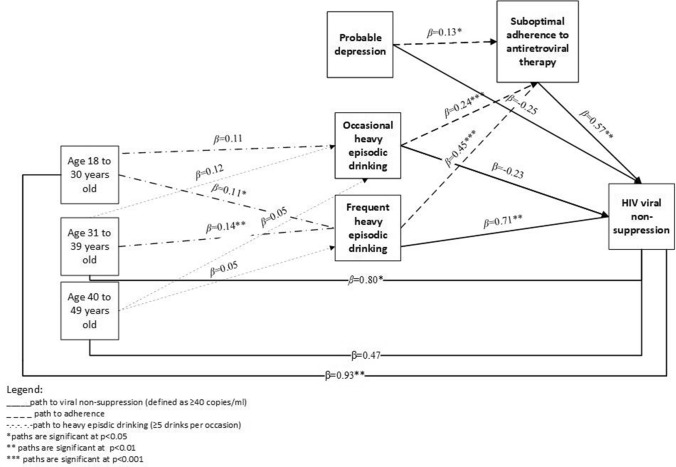
Pathways to HIV viral non-suppression among women living with HIV (n = 608)

None of the age categories were significantly associated with occasional HED (18–30 years old: *β* = 0.11, *SE* = 0.07, *p* = 0.125; 31 to 39 years old: *β* = 0.12, *SE* = 0.07, *p* = 0.823; 40–49 years old: *β* = 0.05, *SE* = 0.06, *p* = 0.436). Being 18–30 or 31–39 years of age was positively associated with frequent HED (*β* = 0.11, *SE* = 0.04, *p* = 0.011; *β* = 0.14, *SE* = 0.04, *p* = 0.001, respectively) and viral non-suppression (*β* = 0.93, *SE* = 0.30, *p* = 0.002; *β* = 0.80, *SE* = 0.33, *p* = 0.024, respectively). Being 40–49 years of age was not significantly associated with frequent HED (*β* = 0.05, *SE* = 0.03, *p* = 0.078) or viral non-suppression (*β* = 0.47, *SE* = 0.27, *p* = 0.101). Significant pathways are displayed in Fig. 1.

Results of the logistic decomposition analysis (Table 5) suggest that a significant part of the relationship between frequent HED and viral non-suppression is the result of a direct effect (OR 2.34, 95% CI 1.63–3.36). The smaller indirect effect suggests that suboptimal adherence only partially mediates the relationship between frequent HED and viral non-suppression (OR 1.24, 95% CI 1.05–1.46). Although the total effect of occasional HED on direct viral non-suppression was non-significant (OR 1.10, 95% CI 0.62–1.62), there was a significant indirect effect (OR 1.11, 95% CI 1.03–1.21), suggesting that this pattern of drinking may indirectly affect viral non-suppression through its relationship with ART adherence.

**Table 5:** Logistic decomposition of the direct and indirect effects of occasional and heavy episodic drinking on HIV viral non-suppression (n = 608)

Model		Direct effect OR (95% CI)	Indirect effect OR (95% CI)	Total effect OR (95% CI)
Effect of drinking patterns^a^ on viral non-suppression mediated by ART adherence	Occasional HED^b^	0.90 (0.57–1.43)	1.11 (1.03–1.21)	1.01 (0.62–1.62)
	Frequent HED	2.34 (1.63–3.36)	1.24 (1.05–1.46)	2.90 (2.05–4.09)

^a^Adjusted for age

^b^Reference category = no/light drinking

*HED* heavy episodic drinking, *OR* odds ratio, *CI* bootstrapped confidence intervals, *ART* antiretroviral therapy

## Discussion

Alcohol consumption is a known risk for suboptimal ART adherence and viral non-suppression, however the pathways through which various drinking patterns affect viral suppression are poorly understood, particularly for WLWH [16, 17]. This study presents some of the first information on specific patterns of alcohol use associated with viral non-suppression and the extent to which adherence to ART mediates these relationships for WLWH. Findings indicate associations between frequent HED and viral non-suppression and clarify that the impact of heavy drinking on viral non-suppression is only partially due to its association with suboptimal ART adherence.

With only two-thirds of participants being virally suppressed and 10% having viral loads indicative of high infectiousness, our results underscore the importance of South Africa implementing a range of additional strategies to improve rates of viral suppression among WLWH to achieve the UNAIDS targets for eliminating HIV by 2030 [41]. In this study, frequent HED was one of the few factors associated with heightened risk of viral non-suppression, doubling the odds of viral non-suppression compared to no/light drinking. Notably, this pattern of drinking was concentrated among younger women who were also most at risk for being virally non-suppressed.

In keeping with Cook et al.’s study [23], our results show that suboptimal adherence only partially mediates the relationship between frequent HED and viral non-suppression. These findings suggest that mechanisms other than adherence account for a substantial part of the relationship between HED and viral load. Other plausible pathways through which heavy drinking may impact viral load include the effect of heavy alcohol use on the immune system (such as increasing inflammatory responses) that worsen disease progression [42, 43] and alcohol’s effect on the metabolism and effectiveness of antiretroviral medicines [44]. As evidence in support of these biological mechanisms is inconclusive, prospective cohort studies of WLWH on ART that examine both the behavioural and biological pathways through which various patterns of alcohol use may impact viral suppression are needed to clarify these relationships. Nonetheless, our results suggest that rates of viral suppression could be improved if evidence-based interventions focused on the detection and reduction of frequent HED are incorporated into HIV treatment services for women. At present, these behavioural health interventions are not part of the HIV treatment service offering within South Africa’s public health system [45].

In contrast, and like studies with men [20, 23], occasional HED was associated with greater risk of suboptimal ART adherence but did not seem to infer greater risk of viral non-suppression relative to alcohol consumption patterns characterised by abstinence or light drinking. Although CASE adherence index scores above the cut-off for optimal adherence correspond to ≥ 95% adherence, many of the newer ART medications retain their effectiveness with 80% adherence [46]. With most study participants being on first line medication, it is possible for women reporting occasional HED to score below the study cut-off optimal adherence yet still be virally suppressed. Even though occasional HED seems to have limited impacts on viral suppression, this pattern of drinking should still be addressed within the context of ART adherence and HIV care—especially for women who may be struggling with adherence. Brief interventions targeting reductions in the volume of alcohol consumed per drinking occasion may help women optimise their adherence to ART and could prevent progression to more frequent HED.

Study limitations need consideration when interpreting these findings. Our sample comprised adult women recruited as part of a trial, of whom 85% met criteria for probable depression. Findings may not be generalisable to the entire population of people on ART in South Africa. Further, we collected self-reported ART adherence and alcohol use data. Social desirability bias may have led to over-reporting of adherence and under-reporting of alcohol use leading to the misclassification of participants into adherence and alcohol use categories. However, studies comparing alcohol self-report and biomarker data for South African women and people on ART have noted low rates of alcohol under-reporting [47, 48]. Nonetheless, future studies should include biological indicators of alcohol use and ART adherence to validate self-report data. Related to this, although we used memory aids to improve participant’s recall of drinking occasions and standard drink estimation, these questions may be affected by recall bias, resulting in measurement error. Future studies could consider combining the collection of these self-report data with the use of technologies such as wearable transdermal alcohol monitors. These have shown promise in facilitating the continuous monitoring of alcohol consumption, although there are some concerns about the ability of these monitors to detect low and moderate levels of alcohol consumption [49]. Although this study did collect other information on alcohol consumption, as reflected through AUDIT-C scores [10], these are relatively blunt indicators of severity of alcohol consumption over a 12 month period providing little insight into various patterns of alcohol consumption that were of interest in this study. For these reasons, we chose not to use AUDIT-C scores in the current analyses. In addition, as analyses were limited to variables included in the MIND baseline assessment, we were unable to examine whether other factors (such as violence exposure and transactional sex) known to be associated with HED among women in this context [50, 51] affected ART adherence and viral suppression in this sample. Inclusion of these additional variables may reveal additional targets for contextually relevant, women-focused alcohol reduction interventions. Finally, data are cross-sectional and causal inferences between frequent HED and viral non-suppression cannot be established. Longitudinal research with a more representative sample of persons on ART is needed to confirm the direction of observed pathways and their clinical significance.

## Conclusion

Study findings have implications for HIV service provision in South Africa and other countries similarly challenged by high rates of alcohol consumption. Alcohol reduction counselling is not routinely provided in the context of HIV care, where alcohol messaging has focused on cessation rather than reduction to within low risk limits [52, 53]. Findings highlight the need to integrate alcohol reduction interventions into HIV care to enable WLWH to reduce the quantity and frequency of their drinking. This is likely to benefit their ART adherence and help them attain viral suppression even if they continue drinking. Findings also point to the potential value of differentiated approaches to alcohol intervention delivery within the context of ART provision, tailored to specific patterns of alcohol consumption. For example, women who report occasional HED may benefit from brief interventions focused on reducing alcohol-related barriers to ART adherence and promoting low risk drinking. In contrast, women who report frequent HED may benefit from extended interventions focused on reducing alcohol use to less harmful patterns while also promoting better ART adherence when drinking.

## Data Availability

Data reported in this paper are available on written request.
